# Transmural left atrial roofline block using rescue cryoballoon ablation after unsuccessful radiofrequency ablation

**DOI:** 10.1016/j.hroo.2025.01.010

**Published:** 2025-01-28

**Authors:** Hiroyuki Kato, Satoshi Yanagisawa, Kazumasa Suga, Hisashi Murakami, Yasuya Inden, Toyoaki Murohara

**Affiliations:** 1 Department of Cardiology, Japan Community Health Care Organization Chukyo Hospital, Nagoya, Japan; 2 Department of Cardiology, Nagoya University Graduate School of Medicine, Nagoya, Japan

**Keywords:** Atrial fibrillation, Cryoballoon, Left posterior wall isolation, Roof, Transmural lesion

**Key Findings**

▪ Achieving a transmural roofline block using radiofrequency catheter ablation (RFCA) remains challenging, as myocardial thickness in the left atrial (LA) roof and the insulation of the septopulmonary bundle by fat interposition can prevent the formation of transmural lesion.
▪ We report a case in which we could not complete the conduction block in LA roof with RFCA but successfully achieved a transmural roofline block using cryoballoon with a detailed evaluation of cryoablation effects on the epicardial bundle.
▪ LA roof ablation by cryoballoon may be a useful rescue treatment option in cases of unsuccessful roofline block using RFCA.

## Introduction

Linear ablation for left atrial posterior wall (LAPW) isolation is a common adjunctive approach to pulmonary vein isolation (PVI) to modify an arrhythmogenic substrate and focal sources in persistent atrial fibrillation (AF). Although previous studies have reported that LAPW isolation was associated with a more significant reduction in AF recurrence compared with PVI alone,^1^^,^^2^ randomized studies have failed to demonstrate a significant improvement in rhythm outcomes after LAPW isolation compared with PVI alone.^3^^,^^4^ One of the reasons for these negative results is probably the inability of radiofrequency catheter ablation (RFCA) to create transmural lesions due to the epicardial muscular bundle (ie, septopulmonary bundle).^5^ Meanwhile, cryoballoon ablation (CBA) to the left atrial (LA) roof exhibited a high success rate of roofline block,^6^^,^^7^ and its possibility of creating transmural lesions in the LA roof has been reported.^8^ Herein, we report a case of successful transmural LAPW isolation by completing a transmural roofline block using CBA as a rescue treatment for unsuccessful RFCA.

## Case report

**Figure 1:**
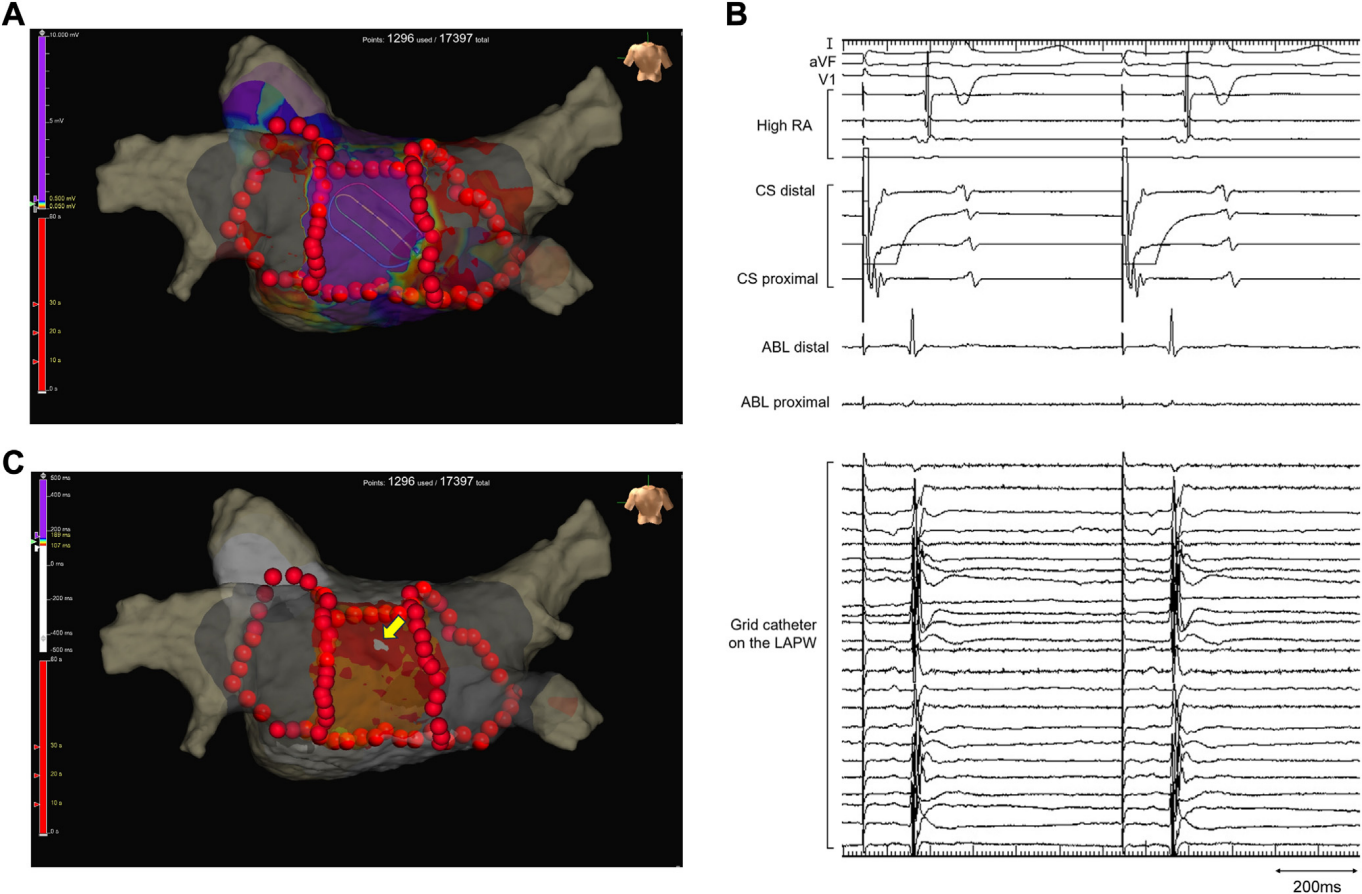
Voltage and activation maps after pulmonary vein isolation and linear ablations. (A) Voltage map during distal coronary sinus (CS) pacing. The purple and gray colors represent voltage amplitudes of >0.5 mV and <0.05 mV, respectively. (B) Atrial electrograms recorded by the grid catheter on the left atrial posterior wall (LAPW). (C) Activation map during distal CS pacing. A centrifugal pattern was observed in the LAPW with the earliest activation site (yellow arrows) below the roofline. This suggests that epicardial propagation via an epicardial bundle crossed the roofline into the LAPW endocardium. Note that the activation propagation did not cross the floorline into the LAPW, indicating that the floorline block was completed. ABL = ablation catheter; RA = right atrium.

**Figure 2:**
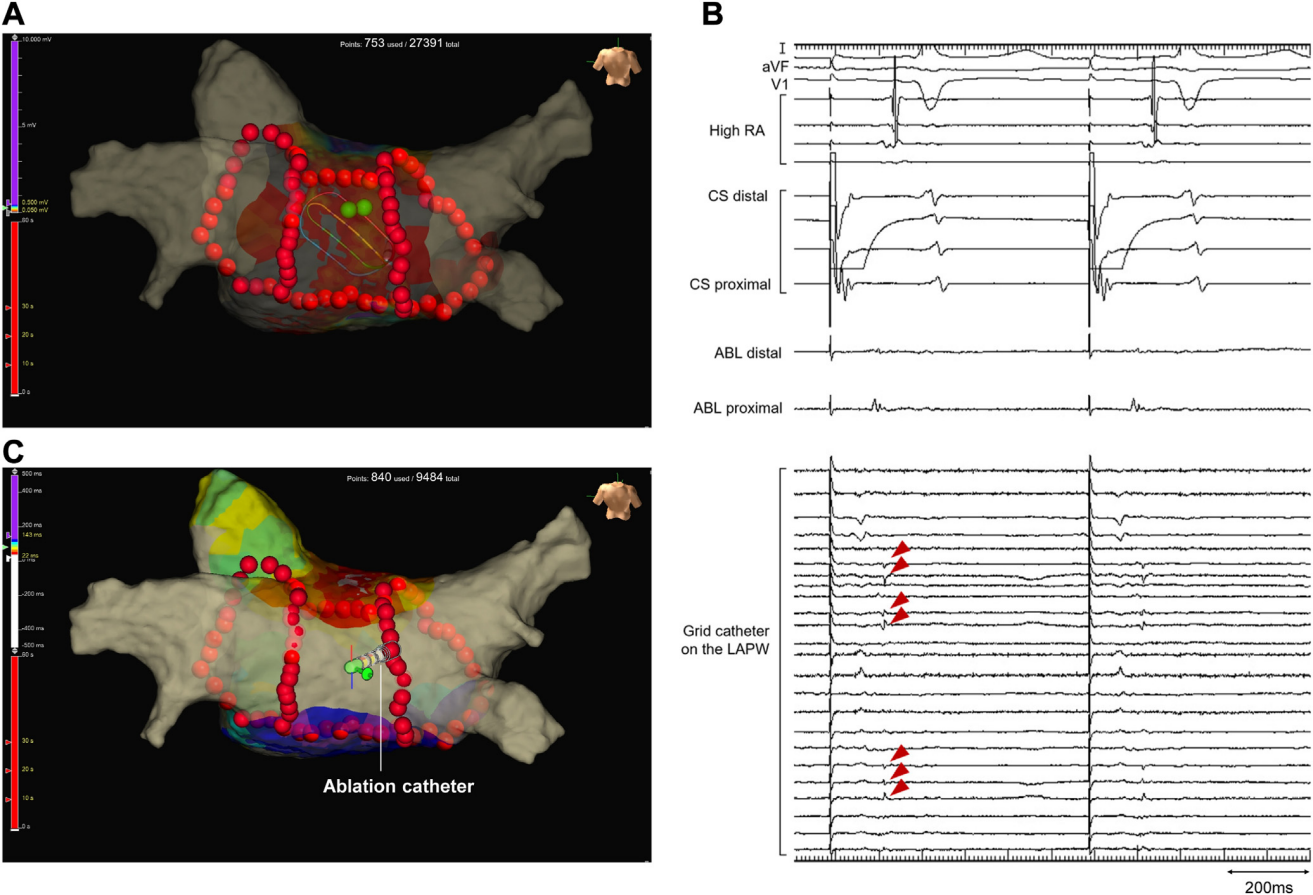
Voltage and activation maps after selective endocardial left atrial posterior wall (LAPW) isolation. (A) Voltage map during distal coronary sinus (CS) pacing. The purple and gray colors represent voltage amplitudes of >0.5 mV and <0.05 mV, respectively. The green tag indicates the ablated site at the earliest activation site. (B) Residual epicardial atrial electrograms (red arrowheads) recorded by the grid catheter on the LAPW. (C) Activation map during ablation catheter (ABL) pacing from the LAPW. The epicardial electrograms in the LAPW could be captured at a pacing output of 20 V at 2.0 ms. Note that the activation propagated across the roofline and broke out above the roofline but not above the floor line. RA = right atrium.

**Figure 3:**
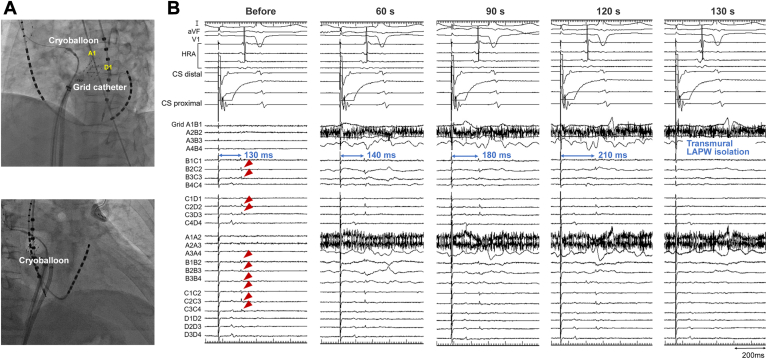
Catheter position and epicardial electrograms on the left atrial posterior wall (LAPW) during cryoballoon ablation. (A) Relationship between the cryoballoon and grid catheter in the left anterior oblique (top) and right anterior oblique (bottom) fluoroscopic views. A1 and D1 indicate the positions of the corresponding electrodes of the grid catheter. (B) Epicardial electrograms (red arrowheads) recorded by the grid catheter on the LAPW during distal coronary sinus (CS) pacing. Before freezing, the interval from pacing to epicardial electrograms on the LAPW was 130 ms. After 60 seconds of freezing, the interval was gradually prolonged. After 130 seconds of freezing, epicardial electrograms were eliminated, indicating successful transmural roofline block and LAPW isolation. HRA = high right atrium.

A 62-year-old man with persistent symptomatic AF refractory to medical therapy was referred to Chukyo Hospital for catheter ablation of AF. The AF was estimated to last for approximately 1 year. Written informed consent was obtained from the patient before the procedure. The ablation procedure was performed under general anesthesia with ventilator management. First, a wide antral circumferential PVI was completed using a TactiFlex ablation catheter (Abbott) with the assistance of an EnSite X 3-dimensional mapping system (Abbott). For PVI, the RF application settings were power of 50 W, contact force of 10 to 20 g, and duration of 15 to 20 seconds. Because AF persisted after PVI was completed, electrical cardioversion was employed to convert AF to sinus rhythm. After recovery to sinus rhythm, several ectopic atrial beats with different activation patterns were observed, some of which were presumed to be originating from the LAPW. Therefore, using point-by-point applications, we added linear ablations to the LA roof and floor for the LAPW isolation. The target settings for linear ablations were power of 50 W, contact force of 10 to 20 g, duration of 20 seconds for the roof and 15 seconds for the floor, and interlesion distance of 4 mm. Because atrial electrograms remained in the LAPW after linear ablations, high-density mappings were created using the Advisor HD Grid mapping catheter (Abbott) during distal pacing of coronary sinus (CS) electrode (Figure 1). The activation mapping in the LAPW indicated a centrifugal pattern with the earliest activation site below the roofline. This suggests that epicardial propagation via an epicardial bundle crossed the roofline into the LAPW endocardium (Supplemental Video 1). RF applications to the earliest activation site could eliminate a high and sharp component of electrograms in the LAPW but not exterminate a small and dull component, indicating the selective endocardial LAPW isolation (Figures 2A and 2B). High-output pacing of 20 V at 2.0 ms from the ablation catheter on the LAPW could capture residual epicardial electrograms, demonstrating retrograde wavefront propagation beyond the roofline (Figure 2C; Supplemental Video 2). Although additional RF applications with a 40 W power and 60-second duration were added to the roofline, they failed to eliminate residual epicardial electrograms in the LAPW. Therefore, CBA using a 28-mm cryoballoon (Arctic Front Advance Pro; Medtronic) was attempted on the LA roof under distal CS pacing while placing the grid catheter on the LAPW. CBA to the LA roof was performed by inserting a circular mapping catheter (Achieve Advance; Medtronic) deep into the right superior pulmonary vein to anchor the cryoballoon.^7^ The cryoballoon was shifted and positioned below the target site on the LA roof by adjusting the orientation of the steerable sheath.^7^ A raise-up technique was employed to facilitate the formation of larger lesions.^9^ After 60 seconds to 130 seconds of freezing, the electrical interval from the pacing to epicardial electrograms on the grid catheter was gradually prolonged. Thereafter, epicardial electrograms were eliminated, indicating successful transmural roofline block and LAPW isolation (Figure 3). The minimum temperature of the cryoballoon was –50 °C during freezing. After 180 seconds of single-shot freezing, no reconnection of the LA roof was observed; however, additional freezings were applied along with the roofline to ensure the durability of roofline lesions. After successful transmural LAPW isolation, the ectopic beat originating from the epicardial LAPW with an exit block to the LA was observed (Supplemental Figure 1). The procedure was completed without any complications. The patient had no atrial arrhythmia recurrences during the follow-up of 6 months.

## Discussion

The achievement of a transmural roofline block by RFCA remains challenging as the LA roof's myocardial thickness and the septopulmonary bundle's insulation by fat interposition can prevent transmural lesion formation.^5^ A previous study showed that 33% of cases required additional “epicardial” RFCA to complete the transmural lesion formation on the roofline.^10^ Transmural LAPW isolation would improve atrial arrhythmia recurrence by eliminating a substrate for endo- and epicardial asynchrony, transmural re-entry, and epicardial focal sources.^11^ Given that the approach of “epicardial” RFCA is not common in AF ablation, our case may be valuable in that we found the usefulness of CBA as a rescue treatment option in cases of unsuccessful transmural roofline block by endocardial RFCA. However, this hypothesis is based on a single case report, and future studies with larger numbers of patients are required to assess the feasibility and durability of transmural lesion formation by CBA on the LA roof.

Although relatively long RF applications with 60-second duration at 40 W power were added to the roofline, they failed to achieve epicardial roofline block. It was unclear whether this unsuccess was due to the thick myocardium in the LA roof wall, edema caused by previous RF applications, or insufficient RF energy. In any case, further, excessive RF applications could not be applied based on the safety profile. Therefore, it would be reasonable to switch to CBA instead of RF applications using aggressive power settings in cases in which creating a transmural lesion by RFCA is difficult.

This case is unique because the intentional precompletion of selective endocardial LAPW isolation allowed a focused and detailed evaluation of the CBA effect on the epicardial bundle in the LA roof. In the present case, the epicardial bundle bypassing the roofline showed a conduction delay at 60 seconds of freezing and a conduction block at 140 seconds of freezing, which were longer than those in the previous report,^8^ indicating that the freezing time required for transmural lesion formation may depend on the patient characteristics. Further investigation is warranted to determine the optimal freezing time of CBA for transmural lesion formation in the LA roof.

CBA for LA roof ablation is considered an off-label use, whereas the utility of roof CBA added to PVI has been reported with accumulated evidence.^7^ Roof CBA is anticipated to yield a high success rate of a roofline block and offer additional benefits, such as arrhythmogenic substrate modifications through debulking of the LAPW and autonomic ganglionic plexi related to the LA dome.^7^^,^^12^ While additional roof CBA did not increase complications compared with PVI using CBA, safety concerns involving injury of the sinus node artery, stiff LA syndrome, and gastroesophageal issues remain for roof CBA.^7^^,^^13^ Pulsed field ablation (PFA) is a novel AF ablation technique that selectively targets myocardial tissue while minimizing damage to adjacent tissues.^14^ A previous study demonstrated that LAPW isolation using a pentaspline PFA catheter for persistent AF was feasible, with no cases of esophageal fistula or other serious complications.^15^ Meanwhile, the efficacy of PFA in creating transmural lesions in the LA roof remains uncertain. If this capability is confirmed, PFA could emerge as a safe and promising alternative to thermal energy ablation for the LA roof.

## Conclusion

The adjunctive approach of CBA could successfully achieve the transmural roofline block and LAPW isolation following an unsuccessful roofline block via RFCA. LA roof ablation using cryoballoon may be a useful rescue treatment option in cases of unsuccessful roofline block via RFCA.

## Disclosures

Satoshi Yanagisawa is affiliated with a department sponsored by Medtronic Japan. The other authors have no conflicts of interest to declare.
