# Supplementary material for: Transmural left atrial roofline block using rescue cryoballoon ablation after unsuccessful radiofrequency ablation
Source: Heart Rhythm O2. 2025 Jan 28;6(4):546–50. doi: 10.1016/j.hroo.2025.01.010 (PMC12047457; doi:10.1016/j.hroo.2025.01.010)
Supplement: Supplemental Data [file mmc1.docx]

**Supplemental appendix**

Kato *et al.*: Transmural left atrial roofline block using rescue cryoballoon ablation after unsuccessful radiofrequency ablation

**Table of contents**

**Page 2:** *Supplemental Figure.* Ectopic beat from epicardial LAPW

**Page 3:** *Supplemental Video legends*

Supplemental Video 1. Sparkle propagation map during distal CS pacing

Supplemental Video 2. Sparkle propagation map during ablation catheter pacing on the LAPW

**Supplemental Figure**

**
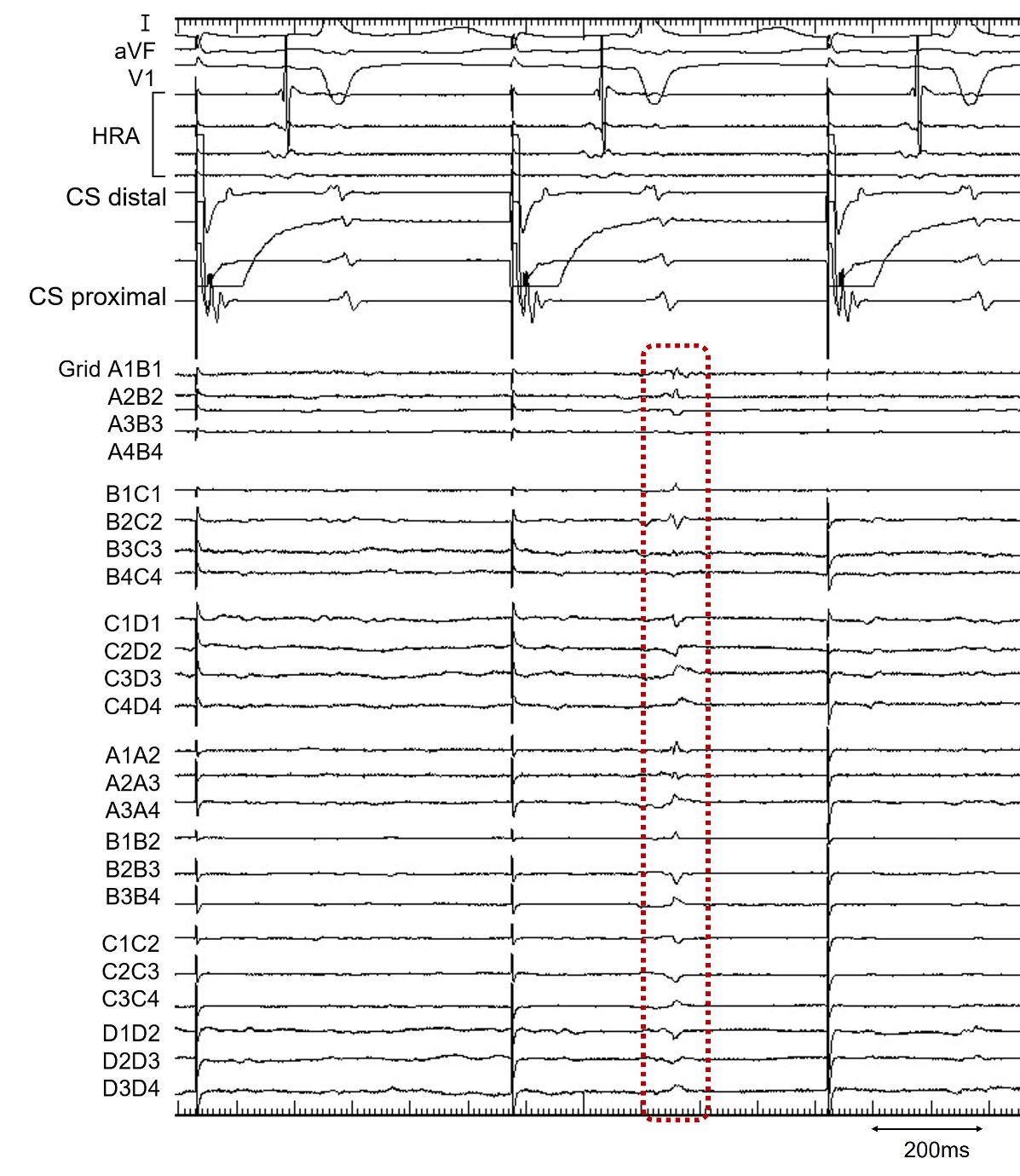
**

**Supplemental Figure: Ectopic beat from epicardial LAPW**

After successful transmural LAPW isolation, the ectopic beat originating from epicardial LAPW with conduction block (dotted red square) was observed.

LAPW, left atrial posterior wall.

**Supplemental Video legends**

**Supplemental Video 1. Sparkle propagation map during distal CS pacing**

The video demonstrates a centrifugal activation pattern with the earliest activation site in the LAPW below the roofline. CS, coronary sinus; LAPW, left atrial posterior wall.

**Supplemental Video 2**. **Sparkle propagation map during ablation catheter pacing on the LAPW**

The video demonstrates activation that propagated across the roofline and broke out above the roofline. CS, coronary sinus; LAPW, left atrial posterior wall.
